# Editorial: Novel Methods for Oncologic Imaging Analysis: Radiomics, Machine Learning, and Artificial Intelligence

**DOI:** 10.3389/fonc.2021.628310

**Published:** 2021-07-22

**Authors:** Hanyue Xu, Lei Deng, Rong Tian, Xuelei Ma

**Affiliations:** ^1^ Department of Biotherapy, West China Hospital, Sichuan University, Chengdu, China; ^2^ Department of Ophthalmology, West China Hospital, Sichuan University, Chengdu, China; ^3^ Department of Medicine, Jacobi Medical Center, Albert Einstein College of Medicine, Bronx, NY, United States; ^4^ Department of Radiology, West China Hospital, Sichuan University, Chengdu, China

**Keywords:** machine learning, radiomics, tumor, artificial intelligence, diagnosis, prognosis

## Introduction

Radiomics is a quantitative and high-throughput radiological method that can aid in clinical decision-making, like treatment modality selection, and treatment plan optimization. By extracting plentiful of parameters from standard images, plenty of information that cannot be discovered by human naked eyes can be explored. Based on the hypothesis that these extra data provide additional information related to gene, protein and tumor phenotype, radiomics has gained increasing attention in cancer research. Meanwhile, because of the rich amount of data obtained in radiomics, sophisticated image analysis tools are required to analyze it. Many image-based signatures have been constructed by computer algorithms. Herein, this Research Topic recruited studies that exploring the usage of radiomics and artificial intelligence assisting clinical decision-making of tumors.

We are very glad to see that many excellent works were submitted to our Research Topic. In the end, a total of 36 papers were published, among which 34 were original studies and two were reviews. The researches were carried out in different countries, including China, USA, UK and France, and most of them were retrospective studies. They used various methods to explore the role of imaging in clinical decision-making. The methods used to select high-throughput imaging parameters can be divided into three levels, including the mathematical formulas level, Machine Learning level and Deep Learning level which belongs to Machine Learning but is more automatic. These kinds of analysis methods are constantly evolving to mimic the thinking patterns of the human brain, gaining the ability to analyze increasingly complicated data. However, for the lack of open platform of images and non-uniform manual feature extraction, there is still a long way to go till a standard or a series of standardized radiomics signatures can be constructed.

## Papers Included in This Research Topic

### Studies With Mathematical Formulas 

As for the first level, using mathematical formulas, some studies generally extract parameters from images, then use statistical methods, like Mann–Whitney U-test, Spearman’s rank correlation test, etc., to compare the internal and external differences of parameters, and then select the most heterogeneous data in different groups. After selecting the appropriate features, Machine Learning algorithms will be used to build models. This kind of studies included in the Research Topic used textures extracted from various images, like Positron Emission Tomography–Computed Tomography (PET/CT), CT, Magnetic Resonance Imaging (MRI), etc., to improve the accuracy of disease differentiation or prognosis prediction (Xu X. et al.; Xu H. et al.; Gao et al.; Hu et al.; Wang J. et al.; Zhou X. et al.; Zhang Y. et al.; Zhong et al.; Wu J. et al.; Zhang P. et al.; Chen W. et al.; Dong Y. et al.; Mai et al.; Li et al.). For instance, Zhang P. et al. differentiated seminomas and nonseminomas by MRI radiomics. Features were selected by comparing their heterogeneity among different groups and by assessing their relevance and redundancy. Then, Least Absolute Shrinkage and Selection Operator (LASSO), a regression analysis method, was used to select features to improve the mode prediction accuracy and interpretability (Zhang P. et al.). Mai et al. focused on the differentiation of phyllodes tumors and fibroadenoma with breast MRI texture analysis. They used a combination of a linear discriminant analysis and the K-Nearest Neighbor classifier to construct differentiative models (Mai et al.).

### Studies With Machine Learning Algorithms

At the second analysis level, studies mainly used Machine Learning algorithms to select and classify radiomics features (Zhou H-F. et al.; Wang F. et al.; Yi et al.; Chen C. et al.; Huang et al.). Machine Learning algorithms build prediction models based on patterns in the training data and make predictions by comparing new instances to previous similar events, and they can be divided into supervised, unsupervised and semi-supervised learning algorithms, based on whether the data are labeled. Some studies selected one kind of algorithms to process data. Fei Wang et al. used LASSO to select features and Support Vector Machine (SVM) algorithm to constructed a predictive model and drew a nomogram to improve the preoperative T category accuracy (Wang F et al.). Huang et al. used LASSO regression model to select features and a multivariable logistic regression to develop predicting models. In addition, a nomogram was drawn by radiomics and clinical features to evaluate peritoneal metastasis status in gastric cancer (Huang et al.). Yi et al. predicted treatment response to neoadjuvant chemoradiotherapy in patients with locally advanced rectal cancer. Three aspects of the treatment response: not only partial clinical remission and good response, but also down-staging were evaluated. They used SVM rather than LASSO or Random Forest (RF) to regress features into a two-dimensional plane (Yi et al.).

Some other studies used multiple methods for feature selection and classification, as there are many kinds of Machine Learning methods with different advantages and drawbacks. They found the choice of classification methods accounted more than selection methods. Chen C. et al. used texture features to differentiate glioblastomas from metastatic brain tumors and differentiate glioblastoma from primary central nervous system lymphoma. In their studies, Linear Discriminant Analysis (LDA)-based models represented better performances than SVM-based models and Logistic Regression (LR)-based models (Chen C. et al.; Chen C. et al.). Similar results were found in other studies that compared different combinations (Tian et al.; Fan et al.; Zhang Y. et al.). For example, Yang Zhang et al. used five selection methods and nine classifiers. The combination of LASSO and LDA represented the best comprehensive performance (Zhang Y. et al.). LDA is a linear classifier whose decision boundary is a plane or a line, while SVM is a non-linear classifier with a decision boundary of a surface or a curved line. Although the above studies showed that LDA was superior to SVM, other studies uncovered the opposite results (Zhang Y. et al.; Payabvash et al.; Delzell et al.; Hong et al.). Zhang Y. et al. differentiated anaplastic oligodendroglioma from atypical low-grade oligodendroglioma. The best-performed combinations were various according to different image parameters. The combination of LASSO and RF classifier was the best for T1 images, while the combination of GBDT and RF classifier was the best for the fluid attenuated inversion recovery images (Zhang Y. et al.). In addition, Payabvash et al. differentiated posterior fossa tumors by using different Machine Learning classifiers, and also found RF models achieved greater accuracy. Delzell et al. used three types of classifying methods, including linear, nonlinear, and ensemble predictive classifying models, and found Elastic Net and SVM performed the best, while RF and Bagged Trees were the worst. It is impossible to draw firm conclusions about which method is the best, because there are too many influencing factors, such as sample size, parameter acquisition, extraction method, etc. However, at the very least, all the relevant articles show that Machine Learning methods are superior to manual methods, so more research on Machine Learning is necessary.

### Studies With Deep Learning

Deep Learning is a subclass of Machine Learning that extends Deep Neural Networks to create complex neural architectures to solve difficult problems which would be impossible with traditional programming based on mathematical logic. Moawad et al. explored the feasibility of volumetric assessment of pre- and post- Transhepatic Arterial Chemotherapy And Embolization hepatocellular carcinoma using fully automated segmentation that based on a Convolutional Neural Network (CNN) approach (U-Net). For automated segmentation, attenuation of adjacent organs and the small size of lesions were the main challenges. According to the assessment of response evaluation criteria in solid tumors, automated segmentation was a good substitute for manual segmentation (Moawad et al.). Sun et al. compared the deep CNN model based on breast ultrasound parameters with the radiomics model. Radiomics can be regarded as an accurate phenotypic analysis of medical images in which the imaging features are carefully defined in advance according to expert opinion. However, Deep Learning uses the raw data and analyzes the pixels and the voxel values by themselves. With convolution techniques, imaging features are automatically defined in the network. Thus, Deep Learning is the most artificial intelligent tool among these three analyzing levels. It is closest to human mode of thinking and can extract features and analyze them automatically.

### Other Related Studies and Reviews

Moreover, there are some included studies focusing on optimizing the original features to promote the analysis results. Lacroix et al. optimized MR images before process with N4ITK bias field correction and normalizing voxel intensities with fat as a reference region. The results showed that correction of magnetic field heterogeneity and normalization of voxel values can promote the usage of radiomic features (Lacroix et al.). Wu W. et al. decomposed data by a non-linear kernelization method, Kernel Principal Component Analysis (KPCA), to find a new set of candidates and maximize the use of data. Lu et al. used concordance correlation coefficients to measure the fidelity in repeated experiments. A lot of features with good repeatability were found and their repeatability can be improved by using specific lesion-drawing methods. Zormpas-Petridis et al. proposed a novel multi-resolution hierarchical framework (SuperCRF) which can introduce the spatial context of a cell as additional information and improve the single cell classification algorithms. In other researches, topics related to radiomics and Machine Learning were discussed. Dong J. et al. and Ge et al. reviewed the usage of radiomics and Machine Learning in the management of cancers, and summarized computer-aided clinical decision-making as a promising solution.

## Conclusion

In conclusion, the combination of radiomics and Machine Learning can provide clinical practice convenience, as long as some obstacles can be solved. The limitations of Machine Learning-based radiological decision-making mainly lie in the following aspects: Firstly, the data quality is uneven, and thus open data-platforms like http://www.predictcancer.org need to be built. Secondly, based on open image sources, algorithms of lesion delineation, feature extraction and signature construction require more standard reference to increase the generalization of the results. Thirdly, more studies that based on uniform data and algorithms and comparing the efficiency of computer-aid and conventional clinical decision-making, are required to better promote the usage of Artificial Intelligence in clinic.

This Research Topic involved many studies, which used the combination of radiomics and Machine Learning in tumor management. We appreciate all the reviewers and authors for their contributions to this Research Topic. We hope this Research Topic can arouse more attention in the related fields.

## Author Contributions

HX and LD wrote the first draft of the manuscript. RT and XM contributed to manuscript revision. All authors contributed to the article and approved the submitted version.

## Conflict of Interest

The authors declare that the research was conducted in the absence of any commercial or financial relationships that could be construed as a potential conflict of interest.

